# DNA vaccination with a gene encoding *Toxoplasma gondii* Rhoptry Protein 17 induces partial protective immunity against lethal challenge in mice

**DOI:** 10.1051/parasite/2016004

**Published:** 2016-02-03

**Authors:** Hai-Long Wang, Yu-Jing Wang, Yan-Jiang Pei, Ji-Zhong Bai, Li-Tian Yin, Rui Guo, Guo-Rong Yin

**Affiliations:** 1 Research Institute of Medical Parasitology, Shanxi Medical University Taiyuan Shanxi 030001 PR China; 2 Department of Biochemistry and Molecular Biology, Shanxi Medical University Taiyuan Shanxi 030001 PR China; 3 Department of General Surgery, Xi’an Red Cross Hospital Xi’an Shanxi 710000 PR China; 4 Department of Physiology, Faculty of Medical and Health Sciences, University of Auckland Private Bag 92-019 Auckland 1142 New Zealand; 5 Department of Physiology, Key Laboratory of Cellular Physiology Co-Constructed by Province and Ministry of Education, Shanxi Medical University Taiyuan Shanxi 030001 PR China

**Keywords:** *Toxoplasma gondii*, Rhoptry protein 17, DNA vaccine, Protective immunity

## Abstract

*Toxoplasma gondii* is an obligate intracellular apicomplexan parasite that affects humans and various vertebrate livestock and causes serious economic losses. To develop an effective vaccine against *T. gondii* infection, we constructed a DNA vaccine encoding the *T. gondii* rhoptry protein 17 (TgROP17) and evaluated its immune protective efficacy against acute *T. gondii* infection in mice. The DNA vaccine (p3×Flag-CMV-14-ROP17) was intramuscularly injected to BALB/c mice and the immune responses of the vaccinated mice were determined. Compared to control mice treated with empty vector or PBS, mice immunized with the ROP17 vaccine showed a relatively high level of specific anti-*T. gondii* antibodies, and a mixed IgG1/IgG2a response with predominance of IgG2a production. The immunized mice also displayed a specific lymphocyte proliferative response, a Th1-type cellular immune response with production of IFN-γ and interleukin-2, and increased number of CD8^+^ T cells. Immunization with the ROP17 DNA significantly prolonged the survival time (15.6 ± 5.4 days, *P* < 0.05) of mice after challenge infection with the virulent *T. gondii* RH strain (Type I), compared with the control groups which died within 8 days. Therefore, our data suggest that DNA vaccination with TgROP17 triggers significant humoral and cellular responses and induces effective protection in mice against acute *T. gondii* infection, indicating that TgROP17 is a promising vaccine candidate against acute toxoplasmosis.

## Abstract (fr)

*Toxoplasma gondii* est un Apicomplexa parasite intracellulaire obligatoire, qui affecte l’homme et divers animaux domestiques et provoque de graves pertes économiques. Pour développer un vaccin efficace contre l’infection par *T. gondii*, nous avons construit un vaccin à ADN codant pour la protéine 17 des rhoptries de *T. gondii* (TRAP17) et avons évalué son efficacité protectrice immunitaire contre une infection aiguë par *T. gondii* chez la souris. Le vaccin à ADN (p3×Flag-CMV-14-ROP17) a été injecté par voie intramusculaire à des souris BALB/c et les réponses immunitaires des souris vaccinées ont été déterminées. Par comparaison avec des souris témoins traitées avec le vecteur vide ou du PBS, les souris immunisées avec le vaccin contre la ROP17 ont montré un niveau relativement élevé d’anticorps spécifiques anti-*T. gondii* et une réponse IgG1/IgG2a mixte avec prédominance de la production d’IgG2a. Les souris immunisées ont également montré une réponse proliférative lymphocytaire spécifique, une réponse immunitaire cellulaire de type Th1 avec production d’IFN-γ et d’interleukine-2, et une augmentation du nombre de cellules T CD8^+^. L’immunisation avec l’ADN ROP17 a prolongé de façon significative le temps de survie (15.6 ± 5.4 jours, *P* < 0.05) des souris après infection d’épreuve avec la souche virulente de *T. gondii* RH (type I), par rapport aux groupes de contrôle qui sont morts dans les 8 jours. Par conséquent, nos données suggèrent que la vaccination par ADN avec TgROP17 déclenche des réponses humorale et cellulaire importantes et induit une protection efficace chez la souris contre une infection aiguë par *T. gondii*, indiquant que TgROP17 est un candidat vaccin prometteur contre la toxoplasmose aiguë.

## Introduction

*Toxoplasma gondii* (*T. gondii*) is an obligate intracellular apicomplexan parasite which can invade a wide range of vertebrate hosts including humans and cause a variety of clinical infections in humans [19, 24]. *T. gondii* infection-induced abortions have been reported mostly in sheep but scarcely in cattle, while it evokes stillbirths and neonatal deaths in all types of livestock with serious economic losses [10]. Infected meat can serve as a source of transmission to humans [3]. *T. gondii* infection thus poses serious public health issues in the world [38]. Currently, there are no drugs available to effectively eliminate the parasite. Therefore, development of an effective vaccine against *T. gondii* infection represents a promising alternative for human health and animal husbandry [44].

Among the putative vaccine candidates for toxoplasmosis, the rhoptry proteins (ROPs) appear to be particularly promising [4, 46]. ROPs are secreted by rhoptries, which are apical secretory organelles of *T. gondii*, and these proteins are involved in parasitic invasion [2, 27]. Some ROPs (i.e. ROP5, ROP16, and ROP18) act as serine-threonine kinases, known as ROP kinases (ROPK), and play the role of virulence factors [11, 15, 40]. ROP17 belongs to the ROP2 superfamily of predicted ROPKs [9]. It possesses a key ATP-binding domain and conserved residues in its catalytic triad (KDD) region [23], and has been proven to be a ROPK [11, 34].

Recently, a number of ROPs including ROP5, ROP9, ROP13, ROP16, and ROP18 have been used as immunogens for vaccine development to evoke considerable cellular and humoral immune responses that partly protected mice against acute infection by *T. gondii* [4, 18, 36, 43, 46]. Our previous study also showed that recombinant rhoptry proteins 17 (rTgROP17) as a candidate protein vaccine could partially protect mice against infection by *T. gondii* via intranasal immunization [35]. However, the protective role of ROP17 as a DNA vaccine has not been tested. In the present study, we constructed the ROP17-expressing eukaryotic expression vector p3×Flag-CMV-14-ROP17 as a DNA vaccine to immunize BALB/c mice, and investigated immune responses and protective efficacy against acute *T. gondii* infection.

## Materials and methods

### Mice, parasites, and recombinant eukaryotic expression plasmid

Female BALB/c mice aged 6 weeks were purchased from the Institute of Laboratory Animal Science of the Chinese Academy of Medical Science (Beijing, China). All mice were maintained under standard, pathogen-free conditions and provided with rodent feed and water ad libitum. All surgeries were performed under sodium pentobarbital anesthesia and all animal experiments were conducted according to institutional guidelines for animal ethics. The tachyzoites of the virulent *T. gondii* RH strain were maintained and collected from the peritoneal cavity of infected BALB/c mice in our laboratory according to a previously described method [20, 41] and used as a challenge for the immunized mice.

The eukaryotic expression vector p3×Flag-CMV-14-ROP17 was constructed and full length ROP17 was expressed (molecular weight, approximately 70 KDa) in HEK 293T cells as in our previous study [34]. Briefly, total tachyzoite RNA was extracted from 5 × 10^8^ tachyzoites using Trizol reagent and the first strand of cDNA was synthesized using the HiFi-MMLV cDNA Kit (CWBIO, China). The coding region of *rop17* of *T. gondii* (1821 bp, which encodes a 607-amino acid protein. GenBank Accession No. AM075203.1) was amplified via polymerase chain reaction (PCR) from the first strand of cDNA. The forward primer was 5′-CGGGGTACCGCCATGGAGTTGGTGTTGTGCTTTGT-3′, the reverse primer was 5′-CGCGGATCCCTCCTTCTGTAATAAAGCCGCCT-3′, containing the *Kpn* I and *Bam*H I restriction sites (underlined), respectively. PCR amplification was performed with initial denaturation at 94 °C for 5 min followed by 30 consecutive cycles of denaturation at 94 °C for 30 s, annealing at 58 °C for 30 s, and extension at 72 °C for 90 s, and then a final extension at 72 °C for 10 min. The amplified products were analyzed by electrophoresis on a 1% (w/v) agarose gel. The p3×Flag-CMV-14 vector and ROP17 PCR products were digested with *Kpn* I and *Bam*H I and then purified from agarose gel using the CWBIO Gel Extraction Kit. The digested p3×Flag-CMV-14 vector and ROP17 PCR products were linked by T_4_ ligase and then transformed into DH 5α host bacteria cells. Positive transformants (p3×Flag-CMV-14-ROP17) were selected and confirmed by DNA sequencing. Recombinant plasmid DNAs were then extracted using GoldHi EndoFree Plasmid Maxi Kit (CWBIO, China) and their concentrations were determined spectrophotometrically. Following dilution with sterile phosphate-buffered saline (PBS) to a final concentration of 1 μg/μL, the recombinant plasmid DNAs were stored at −20 °C until used. The expression of ROP17 was verified by transfecting the p3×Flag-CMV-14-ROP17 plasmid DNA into HEK 293T cells and then detected via Western blot using Flag monoantibody [34].

### Ethics statement and animal experiments

All experimental animal procedures were approved by the Ethics Committee of Animal Experiments of Shanxi Medical University (Permit Number: 20110320-1). Surgeries were performed under sodium pentobarbital anesthesia, and all possible efforts were made to minimize the suffering of the experimental mice according to the protocols from the Laboratory Animal Use and Care Committee of Shanxi Medical University (SXMU-2011-16).

Mice (16/group) were injected intramuscularly (buttocks) with 100 μg of p3×Flag-CMV-14-ROP17 plasmid DNA (in 100 μL sterile PBS; needle length, 16 mm) in the thigh skeletal muscle and boosted twice with the same dose at 2-week intervals. Control mice received PBS alone or empty p3×Flag-CMV-14 vector. Blood was collected from the tail veins of six mice in each group at weeks 0, 2, 4, and 6 and stored at −20 °C until assayed for antibody titers.

Two weeks after the last immunization, six mice per group were sacrificed and splenocytes were harvested under aseptic conditions for cytokine assays and lymphocyte proliferation assays. The remaining mice (10/group) in all groups were intraperitoneally (i.p.) challenged with 1 × 10^3^ *T. gondii* RH strain tachyzoites suspended in 100 μL PBS and their survival periods were recorded daily until all mice died.

### Antibody titers and isotype determination

ROP17-specific antibodies were analyzed by enzyme-linked immunosorbent assay (ELISA) as previously described [35]. In brief, microtiter plates were coated with recombinant TgROP17 protein (rTgROP17, 750 ng/well) in 100 μL carbonate buffer (50 mM, pH 9.6) overnight at 4 °C; nonspecific binding sites were blocked with 5% bovine serum albumin (BSA) in PBS for 1 h at 37 °C. Serum samples diluted in PBS (1:200) were added to the wells (100 μL/well) and incubated at 4 °C overnight. HRP-conjugated goat anti-mouse IgG was used as the secondary antibody, and HRP-conjugated goat anti-mouse IgG1 or IgG2a (Proteintech Group Inc., Chicago) was used for isotype analyses. Immune complexes were revealed by incubation with orthophenylene diamine and 0.15% H_2_O_2_, dark incubated for 30 min and the enzyme reaction was terminated by the addition of 1M H_2_SO_4_. The optical density was read at 492 nm (OD492) with an ELISA reader (Epoch Multi-Volume Spectrophotometer System, BioTek, USA). All samples were run in triplicate.

### Lymphocyte proliferation assay

Spleen cells were collected using published methods [20] and resuspended in RPMI-1640 medium supplemented with 10% fetal calf serum. Cells were seeded in flat-bottom 96-well microtiter plates at a density of 5 × 10^5^ cells per well and were cultured in the presence of rTgROP17 (10 μg/mL), Concanavalin A (Con A; 5 μg/mL; positive control), or RPMI-1640 medium alone (negative control) at 37 °C in a 5% CO_2_ incubator. The proliferative activity was measured using Cell Counting Kit-8 reagent (Dojindo Laboratories; Kumamoto, Japan) according to the manufacturer’s instructions. The stimulation index (SI = the mean OD_450_ values from recombinant antigen-stimulated cultures/the mean OD_450_ values from non-stimulated cultures) of each group was calculated. All assays were performed in triplicate.

### Cytokine assays

Cytokines were measured according to the method described previously [33]. The splenocytes (1.5 × 10^6^) were cultured in triplicate in flat-bottom 24-well microtiter plates and stimulated with 10 μg of rTgROP17. Cell-free supernatants were collected and assayed for interleukin-2 (IL-2) and interleukin-4 (IL-4) at 24 h, for interleukin-10 (IL-10) at 72 h, and for gamma-interferon (IFN-γ) at 96 h. The concentrations of IL-2, IL-4, IL-10, and IFN-γ were determined with a commercial ELISA Kit (NeoBioscience, China) according to the manufacturer’s instructions. All assays were performed in triplicate. The detection limits of the assays were 15.6 pg/mL for IL-2, IL-4, IFN-γ, and 31.25 pg/mL for IL-10.

### Flow cytometry

For phenotypic analysis of splenocytes, a single cell suspension was prepared as described above, and 1 × 10^6^ cells in 50 μL were delivered to each tube already containing 10 μL of Allophycocyanin (APC)-labeled anti-mouse CD4, 20 μL of phycoerythrin (PE)-labeled anti-mouse CD8, or 20 μL of fluorescein isothiocyanate (FITC)-labeled anti-mouse CD3 antibodies (all from eBioscience) and incubated at 4 °C for 20 min in the dark. After washing, the cells were fixed with FACScan buffer (PBS containing 1% BSA and 0.1% sodium azide) and 2% paraformaldehyde. The fluorescence profile of each sample (at least 10,000 cells) was analyzed on FACS-Calibur flow cytometer (BD Biosciences) using SYSTEM II software (Coulter).

### Statistical analysis

Statistical analysis was performed using SPSS software. Normal distribution tests of data within each group were initially determined by the Shapiro-Wilk analysis, and *P* > 0.10 was defined as normally distributed. All results are presented as means ± SD, including antibody levels, lymphoproliferation assays, cytokine productions, and percentages of CD4^+^ and CD8^+^ T cells. Comparisons were made between the different groups by one-way ANOVA and *P* < 0.05 was defined as statistically different. GraphPad Prism 5.0 software was used to construct the survival curve and the differences of survival time were calculated using rank-sum test, as the survival data among different groups were abnormally distributed.

## Results

### Humoral response induced by DNA vaccination

**Figure 1.**
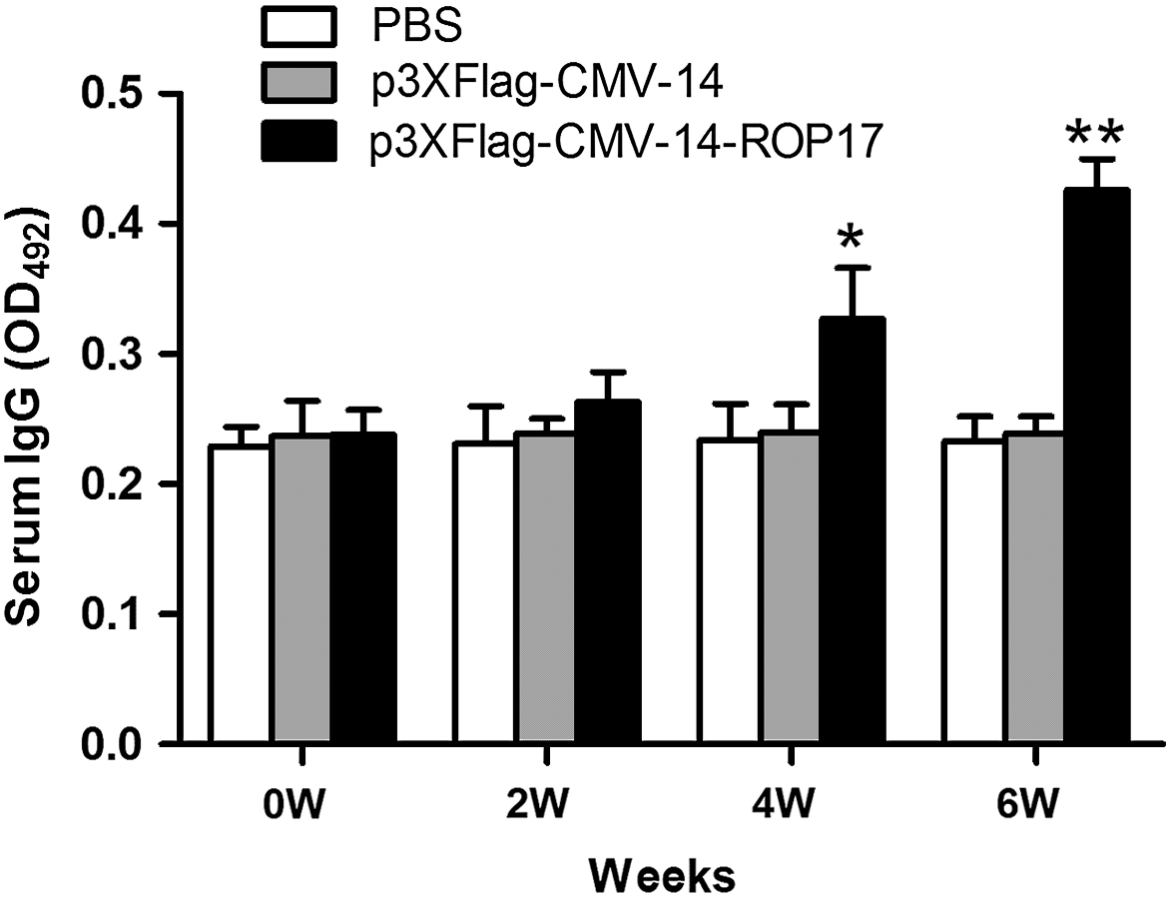
Dynamics of antibody production in ROP17 DNA-immunized BALB/c mice and controls. Levels of specific anti-ROP17 IgG titers in the sera of various BALB/c mice were determined by ELISA under the same conditions. Results are expressed as means ± SD (*n* = 6). *P < 0.05, **P < 0.01 relative to control groups.

To determine the humoral immune responses of the DNA vaccine p3×Flag-CMV-14-ROP17, levels of anti-ROP17 antibodies in all the test animal sera were measured by ELISA. As shown in Figure 1, a significant increase of ROP17-specific IgGs was evident in the mice immunized with p3×Flag-CMV-14-ROP17 for 4 weeks (*P* < 0.05) and reached much higher levels at 6 weeks with successive immunization (*P* < 0.01). In contrast, mice immunized with control p3×Flag-CMV-14 DNAs or treated with PBS had no detectable change of anti-ROP17 antibodies. The antibodies generated in the ROP17 DNA-immunized mice were about twofold those determined in the control mice.

**Figure 2.**
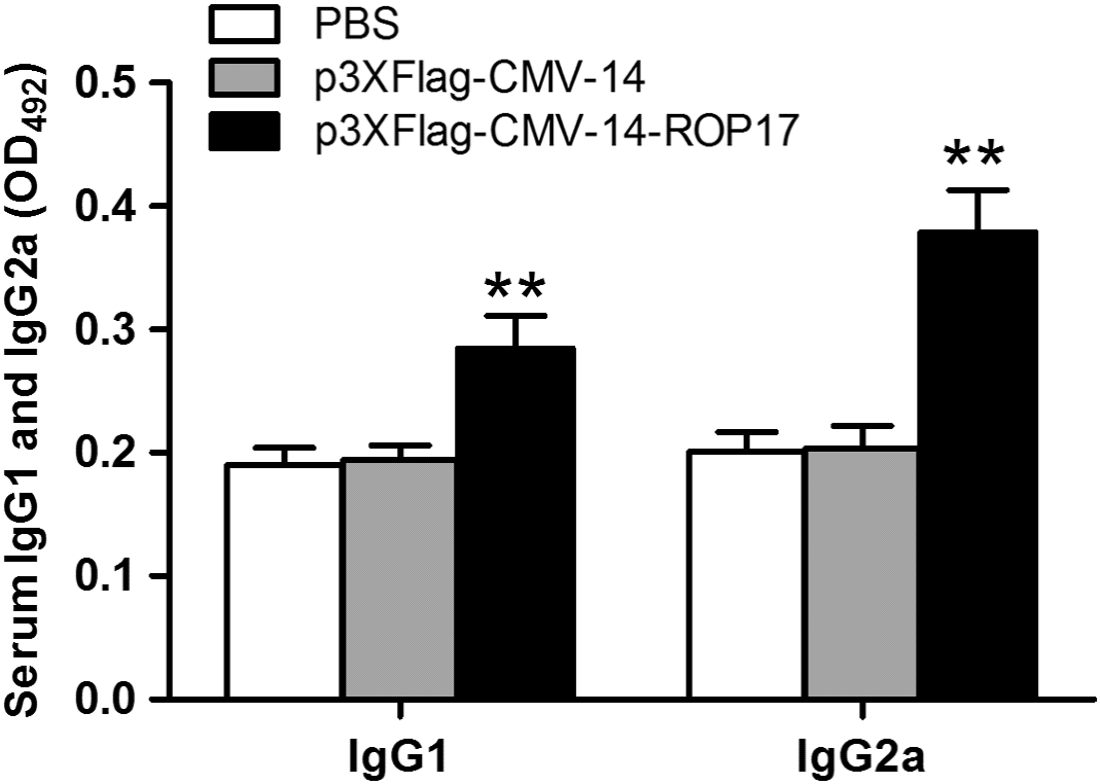
Determination of *T. gondii*-specific IgG subclass titers in ROP17 DNA-immunized BALB/c mice and controls. The IgG subtypes were determined by ELISA as stated in Figure 1. Results are expressed as means ± SD (*n* = 6). *P < 0.05, **P < 0.01 relative to control groups.

We next characterized whether a Th1 or Th2 response was elicited in the immunized mice by evaluating the distribution of IgG subtypes (IgG1 and IgG2a) against ROP17 at two weeks after the last immunization. As shown in Figure 2, ROP17 vaccination induced significant productions of antigen-specific IgG1 and IgG2a antibodies (*P* < 0.05) and a higher level of isotype IgG2a antibodies was observed. These results indicated that ROP17 DNA vaccination could elicit both Th1- and Th2-specific but more Th1-type shifted humoral responses (IgG2a/IgG1 ratio > 1) [22].

### Cytokine production by spleen cells from mice immunized with ROP17 DNA vaccines

To test the possible role of cell-mediated immunity in the mice immunized with the ROP17 vaccines, we analyzed the levels of cytokine (IFN-γ, IL-2, IL-4, and IL-10) productions in the tissue culture media of spleen cells isolated from the immunized animals and stimulated with rTgROP17 proteins. As shown in Table 1, compared to PBS or empty vector controls, spleen cells from p3×Flag-CMV-14-ROP17 immunized mice were able to produce large amounts of IFN-γ and IL-2 (*P* < 0.05) with only slightly increased production of IL-4 and IL-10 (*P* > 0.05). These data suggest that ROP17 DNA vaccination evokes predominately Th1-type cellular immune responses [22, 30].

**Table 1.** Cellular proliferation and cytokine production by splenocytes isolated from ROP17 DNA-immunized BALB/c mice and controls.

Groups*	Cytokine production (pg/mL)**				Lymphocyte SI
	IFN-γ	IL-2	IL-4	IL-10	
PBS	41.29 ± 6.73^a^	79.26 ± 5.35^a^	78.84 ± 4.37^a^	63.31 ± 5.13^a^	1.02 ± 0.11^a^
p3×Flag-CMV-14	45.17 ± 6.93^a^	81.14 ± 5.71^a^	76.63 ± 5.15^a^	66.457 ± 5.26^a^	1.163 ± 0.12^a^
p3×Flag-CMV-14-ROP17	186.17 ± 11.47^b^	158.41 ± 11.38^b^	75.63 ± 2.73^a^	70.25 ± 4.74^a^	2.14 ± 0.23^b^

* *n* = 6 per group.

** Splenocytes were harvested from the mice 2 weeks after the final immunization. Results are presented as means ± the standard errors of three replicate experiments. Values for IFN-γ are for 96 h, values for IL-10 are for 72 h, and values for IL-2, IL-4 are for 24 h. Letter a indicates no statistical difference was observed (*P* > 0.05), and letter b means significant difference within each cytokine or lymphocyte SI group (compared with PBS or empty vector group, *P* < 0.05).

### Cellular proliferative response induced by DNA vaccination

To determine the proliferative immune responses of mice to ROP17 DNA vaccination, splenocytes were harvested 2 weeks after the third immunization from the mice. As shown in Table 1, the splenocyte stimulation indices (SIs) of the mice that were immunized with p3×Flag-CMV-14-ROP17 were significantly greater than those of the control groups (*P <* 0.01). The splenocytes from all experimental groups proliferated to comparable levels in response to ConA, a well-defined stimulator of lymphocyte proliferation [7]. These results further confirm that ROP17 DNA immunization could trigger cell-mediated immunity in mice.

### Phenotypic T lymphocyte induction by DNA vaccination

**Figure 3.**
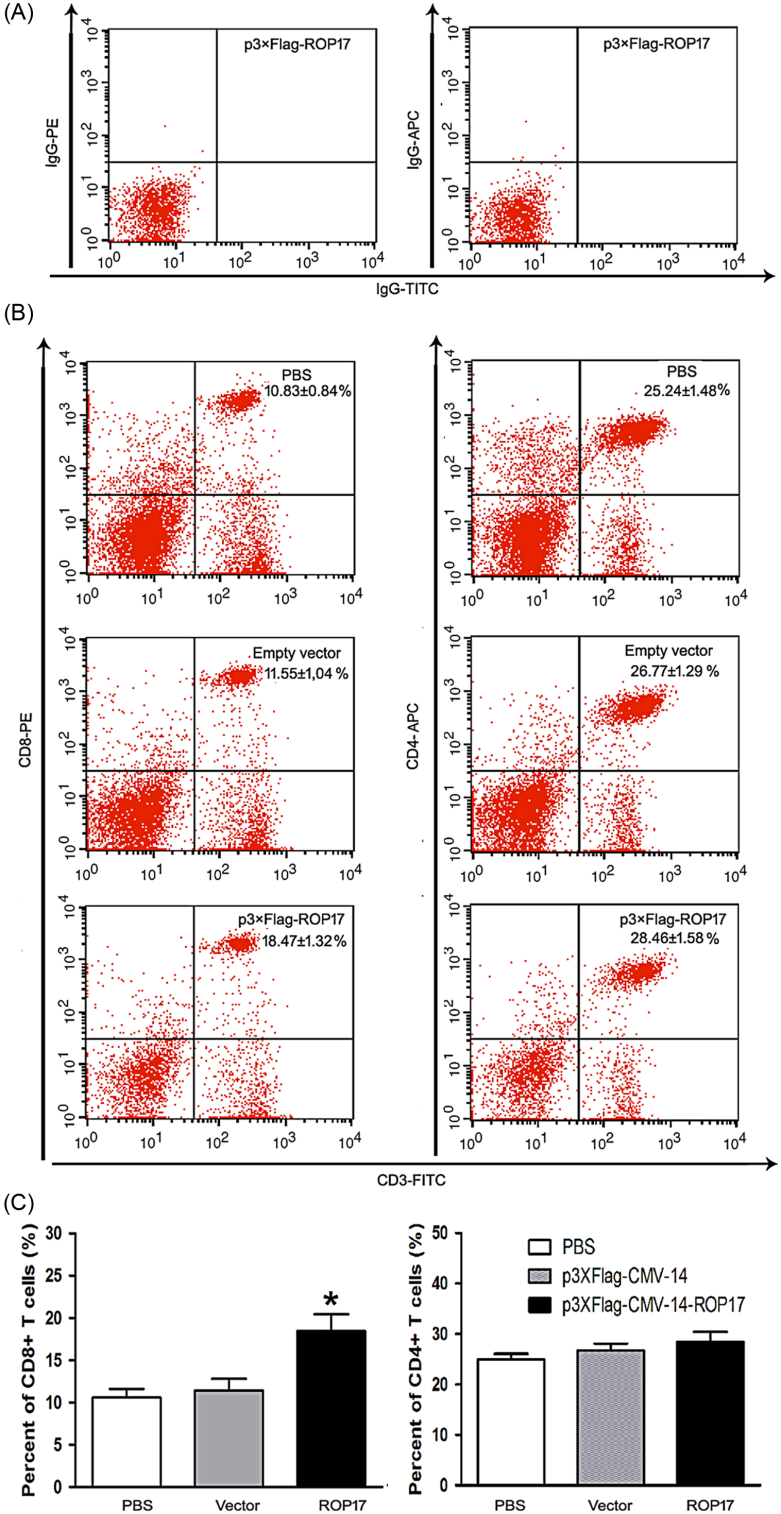
Lymphocyte subpopulations determined in ROP17 DNA-immunized mice by FACS. (A) The total mouse spleen cells were analyzed by using mouse FITC-IgG, PE-IgG, and APC-IgG for quality control. (B and C) The percentages of CD3^+^/CD4^+^ and CD3^+^/CD8^+^ T lymphocytes in the total spleen cells were calculated using flow cytometry analysis. Results are expressed as mean values ± SD (*n* = 6). *P < 0.05 relative to control groups.

To explore whether any specific type of T lymphocyte was involved in the immune response to ROP17 vaccination, FACScanning experiments were performed with fluorescently labeled splenocytes using CD3, CD4, and CD8 antibodies. The total mouse spleen cells were quantified and the percentages of CD3^+^/CD4^+^ and CD3^+^/CD8^+^ T cells in each group were determined. To avoid false positives, we used mouse FITC-IgG, PE-IgG, and APC-IgG to analyze the spleen cells isolated from ROP17-vaccinated mice as a quality control (Fig. 3A). As shown in Figures 3B and 3C, a slightly higher level of CD3^+^/CD4^+^ T lymphocytes was visualized in the ROP17-immunized mice. In contrast, a greater percentage of CD3^+^/CD8^+^ T cells was observed in the ROP17-vaccinated mice than those in the control groups (*P* < 0.05). No difference in the level of these CD3^+^/CD4^+^ and CD3^+^/CD8^+^ T-cell subtypes was seen between the two control groups (*P* > 0.05). These data suggest that ROP17 DNA vaccination-induced immunity is also CD8^+^ T cell-mediated in mice.

### Protective efficacy of DNA vaccination against *T. gondii* acute infection in mice

**Figure 4.**
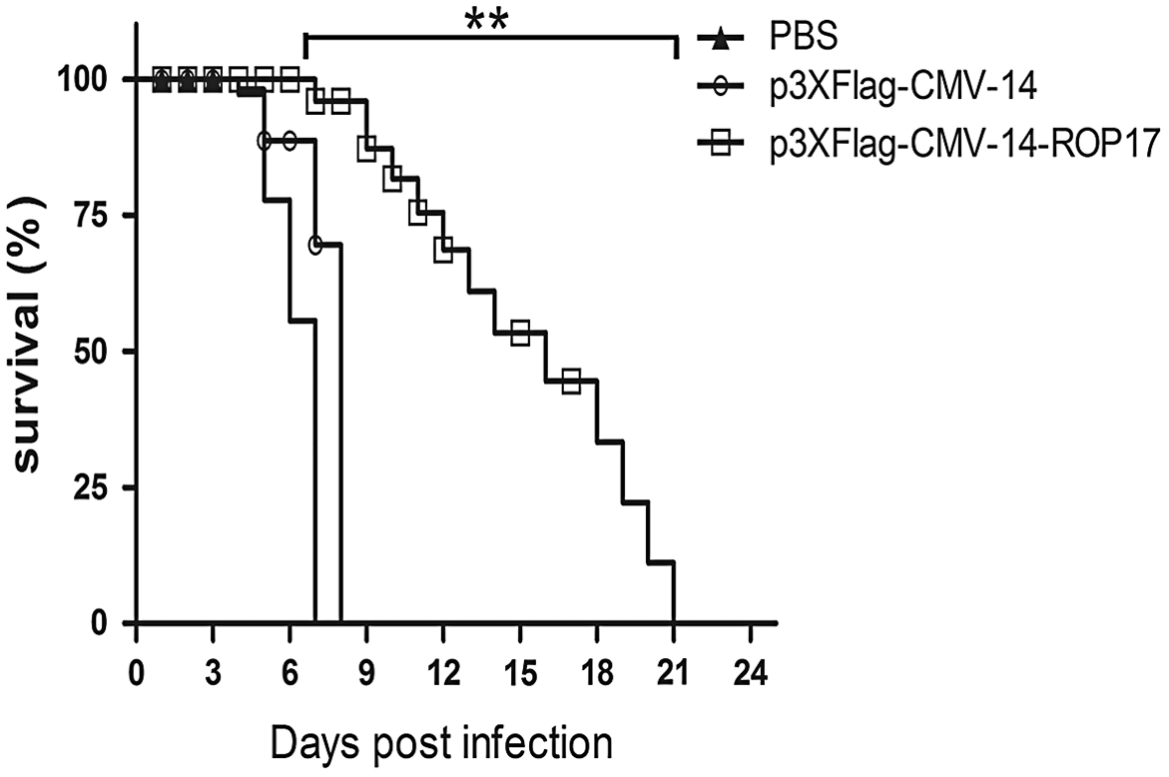
Protection of BALB/c mice against *T. gondii* acute infection. Survival curves of ROP17 DNA-immunized and control BALB/c mice were determined following lethal challenges with 1000 tachyzoites of virulent *T. gondii* RH strain at 2 weeks after the last immunization. Results are representative of two independent experiments and values are presented as means ± SD (*n* = 10). **P < 0.01 relative to the control groups.

To assess the protective immunity of p3×Flag-CMV-14-ROP17, mice were challenged with 1 × 10^3^ tachyzoites of the RH strain at 2 weeks after the final immunization. As the RH virulent strain is non-cystogenic [8, 32], we determined survival rather than tissue cyst burden in the infected mice to assess the protective efficiency of the DNA vaccine. As shown in Figure 4, the survival periods of the mice that were immunized with the ROP17 vaccine ranged from 7 to 21 days, while the control mice died between days 4 and 8 after the challenge (*P* < 0.01). These results indicated that immunization with ROP17 DNAs could prolong survival in BALB/c mice.

## Discussion

Various *T. gondii* rhoptry proteins, such as ROP5, ROP8, ROP9, ROP13, ROP16, ROP18, and ROP38, have been evaluated as potential vaccine candidates for toxoplasmosis. Here we show that ROP17 is also a potential vaccine candidate. ROP17 DNA vaccination prolongs the survival of mice for 15.6 ± 5.4 days following a challenge infection with tachyzoites of the RH strain (Fig. 4). Previous DNA vaccination trials also gave a similar mice survival time of 24.9 ± 2.3 for ROP13 and 21.6 ± 9.9 days for ROP16 [36, 42]. Similar immunization studies indicated that longer mice survival was achievable with ROP8 (29 days) and ROP18 DNAs (27.9 ± 15.1 days) [21, 43], whereas shorter protection of mice was obtained with ROP9 (12.9 ± 2.9 days) and ROP38 DNAs (8.1 ± 0.75 days) [4, 39]. These data together suggest that ROP17 DNA vaccination can confer a high degree of immune protection in mice.

Generally, specific-IgG antibodies against *T. gondii* can prevent the parasite from attaching to its host cell receptors and promote macrophages to kill intracellular parasites, which is important in controlling *T. gondii* infection and preventing reactivation [26]. In the present study, an elevated level of anti-ROP17 antibodies was detected in mice immunized with ROP17 DNAs in comparison with those of their control groups (Fig. 1). A mixed IgG1/IgG2a response but a predominant production of IgG2a was also observed in mice immunized with ROP17 vaccine but not in the empty vector or PBS controls (Fig. 2). Together, these data indicate the involvement of a Th1-type shift of both Th1- and Th2-specific humoral responses during ROP17 DNA vaccination.

It is well known that T cell-mediated adaptive immune responses are important to determine the course of *T. gondii* infection [16]. In the present study, a significant proliferative response of splenocytes was detected following ROP17 DNA immunization, indicating the activation of cellular immune responses. Additionally, ROP17 DNA vaccination also significantly boosted the percentage of CD8^+^ T cells, whereas the number of CD4^+^ T cells was similar to that of the control groups (Fig. 3). Although this CD8^+^ response may be lower than that from natural infection [25], this is consistent with the notion that CD8^+^ T cells constitute the major cellular T cell subset which is involved in acquired immune protection against *T. gondii* [12]. Therefore, our data suggest that ROP17 DNA vaccination-induced cellular immune responses involve a specific population of CD8^+^ T cells.

In agreement with the above observations, elevated levels of both IFN-γ and IL-2 cytokines were detected in isolated spleen cells from the ROP17-immunized mice (Table 1), suggesting a Th1-type immune response. The finding of slightly increased production of IL-4 and IL-10 from spleen cells of the ROP17-immunized mice (Table 1) further indicates a possible role of Th2-type response as well [23, 24]. A Th1-dominated immune response is consistent with the observation of increased IgG antibody subtypes and high ratio of IgG2a to IgG1 antibodies in the mice immunized with ROP17 DNA vaccines (Figs. 1 and 2). This is supported by the findings that Th1-type immune response plays a critical role in protective immunity against *T. gondii* [28], and that Th2-type immune response is required during the early phase of acute *T. gondii* infection [1]. Taken together, all these data support a role for Th1-type dominated and both humoral and cell-mediated immune responses in the mice immunized with the ROP17 DNA vaccines.

To determine the protective effect of ROP17 DNA vaccine against toxoplasmosis, we used the virulent *T. gondii* RH strain because it causes severe damage in animals and has been widely used to assess the protective efficacy of novel antigens against toxoplasmosis [5, 13, 37]. As ROP17s across the three genotypes of *T. gondii* share over 99% of amino acid sequence identity as determined in our previous study [34], although there were A + T contents that varied from 49.45% to 50.11% and nucleotide polymorphisms at 33 positions among strains from different hosts and geographical locations [45], a protection test against type II or III *T. gondii* by the ROP17 DNA vaccine was not included in this study. In our previous studies, recombinant *T. gondii* ROP17 protein (rTgROP17) has been used as a vaccine candidate to elicit protective immunity against acute *T. gondii* infection in mice. This protein vaccine induced both systemic and local immune responses and provided a 50% increase in survival rate and longer survival time [35]. Compared to the ROP17 protein vaccine, ROP17 DNA vaccine in the present study provided limit protection against acute *T. gondii* infection. One possible reason for this difference may be the low levels of antibody generated in the ROP17 DNA-immunized mice, being only twice as high as those in control mice, and lower than those elicited by rTgROP17 vaccines. Since IgG-dependent phagocytosis, cytotoxicity, or complement-mediated lysis are crucial mechanisms for resistance to tachyzoites [26], the relatively low levels of antibodies produced during the ROP17 DNA immunization might not be sufficient to prevent acute infections. Likewise, low levels of IL-4 and IL-10 cytokines raised in the ROP17 DNA-immunized mice (relative to the control animals) may not promote sufficient mast cell responses which play an important role in modulating acute inflammatory pathogenesis and parasite clearance during *T. gondii* infection [14]. This is consistent with the relatively low levels of specific-IgG antibodies found in these mice. In addition, relatively low level of CD8^+^ response compared with those in natural infection with a non-lethal strain [29], and a low level of CD4^+^ T response in these ROP17 DNA-immunized mice might be another reason for the low level of protection against acute *T. gondii* infection in the present study because only the synergy between CD8^+^ and CD4^+^ T cells can provide efficient protection against *T. gondii* [6]. Finally, the route of vaccine administration by intramuscular ROP17 DNA injection may also explain its limited protective effects, whereas intranasal rTgROP17 immunization induces strong secretory IgA (SIgA) immune responses in mucosal sites where SIgA serves as the first line of defense in protecting the intestinal epithelium from enteric toxins and pathogenic microorganisms like *Toxoplasma* [31].

In conclusion, our results demonstrate that immunization with ROP17 DNA vaccines evokes both humoral and cellular but Th1-dominated immune responses and prolongs the survival time of these mice upon acute *T. gondii* infection. Despite the partial protective efficacy of the DNA vaccine, ROP17 appears to be a potential candidate for the development of vaccines against *toxoplasmosis*. The immune efficacy of this ROP17-based DNA vaccine may well be improved through its combination with other effective antigens such as ROP18 or adaptive adjuvants such as IL-12 and IL-15 [17].
